# Increased growth response of strawberry roots to a commercial extract from *Durvillaea potatorum* and *Ascophyllum nodosum*

**DOI:** 10.1007/s10811-017-1387-9

**Published:** 2018-01-10

**Authors:** Scott W. Mattner, Mirko Milinkovic, Tony Arioli

**Affiliations:** 1 Victorian Strawberry Industry Certification Authority, Toolangi, VIC 3777 Australia; 2 Seasol International, Bayswater, VIC 3155 Australia

**Keywords:** *Fragaria* × *ananassa*, Root length density, Seasol®, Seaweed extract, Specific root length, Strawberry runner

## Abstract

The withdrawal of soil fumigants like methyl bromide is forcing strawberry growers to consider supplementary and alternative ways of producing crops. In addition to controlling soil-borne pests, soil fumigation causes an increased growth response in strawberry roots, and the use of biostimulants may offer an alternative to replace this response. We tested the hypothesis that treatment with a commercial extract (Seasol®) from the seaweeds *Duvillaea potatorum* and *Ascophyllum nodosum* can increase root growth, and transplant (runner) and fruit yields of strawberry. From 2014 to 2016, we conducted three field trials on strawberry farms in the nursery sector at Toolangi and in the fruiting sector at Coldstream in Victoria, Australia. We applied the seaweed extract as a monthly drench (10 L ha^−1^) to two cultivars of strawberry (‘Albion’ and ‘Fortuna’), compared with an untreated control. In the nursery sector, use of the extract significantly increased the density of secondary roots (feeder roots) on harvested runners by up to 22%. Treatment with the extract also significantly increased yields of marketable runners by 8–19%. In the fruit sector, use of the extract significantly increased the root length density (root length per volume of soil) of strawberry plants by 38% and marketable fruit yields by 8%. Root length density at final harvest and marketable fruit yield of strawberry were highly correlated (*r* = 0.94). This relationship provides an insight into the mode of action of seaweed extracts and is discussed. Overall, the results show the potential benefits of the integrated use of seaweed extracts in strawberry production across the nursery and fruit sectors, and their promise for supplementing or replacing the increased growth response provided by soil fumigants.

## Introduction

The Australian strawberry (*Fragaria* × *ananassa*) industry was recently valued at AUS$420 million per annum. Production occurs in every Australian state, with major regions in Victoria and Queensland. The fruit sector in Victoria predominantly uses day-neutral cultivars of strawberry (e.g. Albion), which fruit in response to mild temperatures over summer. In contrast, the fruit sector in Queensland predominantly uses short-day cultivars of strawberry (e.g. Fortuna), which fruit in response to short days and mild temperatures through winter. Pathogen-tested runners (bare-rooted transplants) form the backbone of a profitable strawberry fruit industry. Seventy percent of Australian runners are produced in Victoria, with the remainder mostly produced in Queensland. In the 2014/2015 season, 90 million runners were planted in the fruit sector (Horticulture Innovation Australia 2017).

Strawberry runner and fruit production in Australia and many regions around the world relies on the use of fumigants to control soil-borne pathogens, weeds and other pests (Ajwa et al. 2003; Fennimore et al. 2008; López-Aranda 2014; Mattner et al. 2014). Soil fumigation can also cause an increased growth response in the roots of strawberry and other crops that is not related to control of soil-borne pathogens and pests (Wilhelm and Paulus 1980; Yuen et al. 1991; Porter et al. 2005). The increased growth response of crop roots is associated with changes in soil biology and chemistry, particularly nitrogen, following fumigation (Ridge 1976; Rovira 1976; Porter et al. 2005). Furthermore, Yuen et al. (1991) found that improved root density and health of strawberry plants caused by soil fumigation was correlated with increased fruit yields. Many soil fumigants are being withdrawn internationally due to their environmental impact (López-Aranda et al. 2016), and strawberry growers need to consider supplementary or alternative practices for improving root growth and health (Porter et al. 2005).

It is well established that application of seaweed extracts to soil and/or foliage can increase root growth and development of plants (Khan et al. 2009; Arioli et al. 2015). This can occur through improved lateral root formation (Artzamon and Staden 1994), increased root initiation (Alam et al. 2013), greater root elongation (Rayorath et al. 2008; Arioli et al. 2015) and increased biomass (Spenelli et al. 2010; El-Miniawy et al. 2014). Researchers have postulated that the stimulatory effect of seaweed extracts on root and plant growth is due to the complex of hormones and other compounds they contain, which act directly or by influencing gene regulation in the plant (Khan et al. 2009; Arioli et al. 2015). For example, Xu et al. (2003) and González et al. (2013) reported that various oligosaccharides contained in seaweed extracts can directly stimulate root growth.

Studies by Spenelli et al. (2010) and El-Miniawy et al. (2014) found that application of biostimulant products containing extracts from the seaweed *Ascophyllum nodosum* increased root dry weight of strawberry by 35–130%, and this corresponded with an increase in fruit yield of 23–30%. Furthermore, Alam et al. (2013) showed that application of a soil amendment containing *A. nodosum* increased the number of roots, total root length, root surface area and total root volume of strawberry plants. However, there are no reports on the effect of seaweed extracts on strawberry root growth and yield in the nursery sector. Furthermore, no studies have evaluated the carry-over effect of seaweed extracts applied in the nursery to runners and strawberry plants in the fruit sector.

We conducted field trials in the strawberry nursery and fruit sectors to test the hypothesis that a commercial extract from *Durvillaea potatorum* and *A. nodosum* (Seasol®) could increase root growth and runner and fruit yields of strawberry.

## Materials and methods

### Strawberry nursery trials

Two field trials were conducted in the nursery sector at Toolangi, Victoria, Australia, in 2014/2015 with the strawberry cultivars ‘Fortuna’ and ‘Albion’. Soils at the trial sites were brown ferrosols, with a clay–loam texture, which is the main soil type occurring in the district. Soils were rotary-hoed to a depth of 25 cm and fumigated with a mixture of methyl bromide and chloropicrin (50:50), 500 kg ha^−1^ in May 2014. In October 2014, beds were marked out (1.73 m apart) and a single row of strawberry mother plants were planted by hand in the beds (spaced 100 cm apart in the Fortuna trial and 50 cm apart in the Albion trial). Before planting, strawberry mother plants were soaked overnight in a 1:400 solution (dilution recommended by the manufacturer) of the seaweed extract or water (untreated control), which is a common industry practice. Two bands of a fertiliser blend (Pivot 800; Incitec Pivot, Portland, Victoria, Australia) were chiselled to a depth of 10 cm on either side of the transplant row at a rate of 900 kg ha^−1^. Top dress applications of fertilisers (Pivot 800 and Calcium Nitrate GHG; Campbells, Laverton, Victoria, Australia) were applied twice through the season (at rates of 900 and 50 kg ha^−1^, respectively). Water was applied by overhead sprinklers using the growers’ irrigation schedules. Runner plants were regularly de-flowered and daughter plants pinned into soil to encourage root production (approximately monthly), and plants sprayed with a rotation of registered pesticides to control mites, aphids, leaf hoppers, powdery mildew, grey mould and leaf blotch.

The trials were conducted as randomised complete block designs, with 16 blocks. Treatments consisted of a seaweed extract and an untreated control. The seaweed extract used in the trials was an alkaline hydrolysis product from *D. potatorum* and *A. nodosum* (Seasol®; Seasol International, Bayswater, Victoria, Australia). The product contains > 50% extract from *D. potatorum* with a soluble solid level of 16% (*w*/*w*) (Arioli et al. 2015). The seaweed extract was applied as a monthly drench (1:400 concentration) over the experimental plot and strawberry plants (including the foliage) at a rate of 10 L ha^−1^. Equivalent volumes of water were applied as the control. The first application was at planting and the last application was 2 weeks before harvest (March 2015 in the Fortuna trial and June 2015 in the Albion trial). In total, there were six applications of the seaweed extract in the Fortuna field trial (i.e. a total 0 (control) and 60 L of the seaweed extract ha^−1^) and eight applications in the Albion trial (i.e. a total of 0 (control) and 80 L of the seaweed extract ha^−1^). Individual plots were 5 m in length (an area of 8.65 m^2^) with 1-m guards between plots.

At final harvest (March 2015 and June 2015 for the Festival and Albion trials, respectively), all runners were dug out from a 1-m linear section of row from each plot. Runners were graded into firsts (runners with a crown diameter > 7.5 mm and a well-formed root system) and rejects as per commercial practice. Runners were harvested with leaves-on in the Fortuna trial and leaves-off in the Albion trial, which is the commercial standard for these cultivars. Following harvest, Albion runners were placed in cold storage (− 2 °C) until planting in a separate trial in the strawberry fruit sector (see below).

Parameters of runner quality (leaf number, area of the most expanded leaf, petiole length, crown diameter, root length, feeder roots score and density of feeder roots) were measured on a random subsample of ten harvested runners per plot. Petiole length, crown diameter and root length were measured with digital callipers, while the area of the most expanded leaf was determined with an area meter (Li-Cor LI300C with transparent belt accessory, USA). To determine the density of feeder roots (secondary roots), five randomly selected structural roots (primary roots) were cut from each runner. A 5 cm length, subsection of root was cut from the middle of each structural root. The number of feeder roots on each subsection of structural root was counted using a dissector microscope. Feeder roots scores were assessed using the scale described by Wing et al. (1995).

Soil samples were taken from each plot at planting and harvest, and analysed for soil nutrients through a commercial laboratory (Nutrient Advantage, Werribee, Victoria, Australia). In this procedure, 20 soil cores (2.5 cm diameter) were taken to a depth of 10 cm from each plot. Soil was thoroughly mixed and a subsample (500 g) taken for analysis.

### Strawberry fruit trial

A field trial was conducted in the strawberry fruit sector at Coldstream, Victoria, Australia, in 2015/2016 with cold-stored runners (cultivar Albion) sourced from the strawberry nursery trial (see above). Soil at the site was a sodosol, with a clay–loam texture. Soil was prepared by rotary hoeing to depth of 25 cm and raising beds. In November 2015, soil was fumigated with chloropicrin (340 kg ha^−1^) and beds covered with black plastic (low-density polyethylene). During soil fumigation, two bands of a fertiliser blend (Pivot 800) were chiselled into soil to a depth of 10 cm at a rate of 800 kg ha^−1^ and trickle-irrigation tape laid under the plastic (two lines per bed).

Prior to planting, half the runners were soaked overnight in a 1:400 concentration of the seaweed extract and half in water (control). In December 2015, runners were planted by hand into plots through holes in the plastic (ca. 10-cm diameter). Beds were 1.02 m wide and there were four rows of strawberries per bed. Individual plants were spaced 40 cm apart. Plants were watered by regular overhead irrigation during establishment (ca. 1 month), after which plants were mostly irrigated by drip irrigation through the trickle tape. All other agronomical procedures in the trial followed standard industry practices.

Half of the plots and plants in the trial were treated with the seaweed extract (1:400 concentration) as a monthly drench (10 L ha^−1^) over the plant and soil in the planting holes. The other half of plots and plants were treated with equivalent volumes of water (control). The first application was at planting and the last application was 2 weeks (June 2016) before final harvest. In total, there were seven applications of the seaweed extract in the field trial (i.e. a total 0 (control) and 70 L of the extract ha^−1^). The trial was conducted as a randomised factorial (2 × 2 = 4 treatments) design with three blocks (20 plants per plot). Individual plots were 2 m in length (an area of 2.04 m^2^), with an 80-cm guard between plots. Treatments included (1) application of the seaweed extract in the nursery industry (two levels—the seaweed extract applied monthly in the nursery (2014/2015) or untreated (water)) and (2) application of the seaweed extract in the fruit industry (two levels—the seaweed extract applied monthly in the fruit sector (2015/2016) or untreated (water)).

Total and marketable fruit were counted and weighed one to three times per week from January to June 2016. Revenue from strawberry fruit for each pick was calculated from national wholesale prices for strawberry fruit (FreshLogic, Hawthorn, Victoria, Australia). At final harvest, soil cores (100 cm^3^) containing strawberry roots from one plant (to a soil depth of 20 cm) were taken (five per plot) using the method described by Yuen et al. (1991). All roots contained in the core samples were washed free from soil and placed into zip-lock polyethylene bags, quarter filled with water and sealed. Images of the roots in the bag were taken with a digital scanner. The total root length in the samples was calculated from the images using Sigmascan Pro 5.0 (Systat Software Inc., USA). Root samples were then oven dried at 80 °C for 4 days and their mass measured. Results were expressed as root length density (root length per volume of soil) and specific root length (root length per root dry weight).

Soil samples were taken from each plot at planting and at final harvest, as described previously, and analysed for soil nutrients through a commercial laboratory (Nutrient Advantage, Werribee, Victoria, Australia).

### Statistical analysis

Data from the trials were statistically analysed using ANOVA on Genstat 18th ed. (VSN International). Homogeneity of variance was determined by examining plots of fitted values versus residuals, while histograms of residuals were examined for normality of distribution. Where variance was heterogeneous across treatments, appropriate data transformations were made to restore homogeneity. Fisher’s least significant difference (LSD) test was used to identify differences between treatment means. The level of significance used was *p* ≤ 0.05. Linear regression was used to test the relationships between root growth and strawberry runner and fruit yields.

## Results

### Strawberry nursery trials

Application of the seaweed extract significantly increased runner yields of Fortuna and Albion cultivars of strawberry by 19 and 8%, respectively, compared with the untreated control (Table 1). Use of the seaweed extract significantly decreased the percentage of rejected runners of the Fortuna cultivar, but not the Albion cultivar (Table 1).

**Table 1:** Commercial runner yields of two cultivars of strawberry (Fortuna and Albion) treated with a seaweed extract from *Durvillaea potatorum* and *Ascophyllum nodosum* (SE) in a trial in the nursery sector at Toolangi, Victoria, Australia

Treatment	Fortuna		Albion	
	Runner yield (runners m^−1^)	Rejects (%)	Runner yield (runners m^−1^)	Rejects (%)
Untreated	86.7 b	43.7 a	144.5 b	11.4 a
SE	103.4 a	35.7 a	156.7 a	10.1 a
LSD (*p* = 0.05)	9.1	5.9	4.8	1.9

Values followed by different letters in each column are significantly different, where *p* ≤ 0.05

Treatment with the seaweed extract significantly increased feeder roots scores and the density of feeder roots on harvested runners of the Fortuna cultivar by 22%, but not the Albion cultivar (Tables 2 and 3, respectively). The extract also increased the crown diameters of harvested runners of the cultivar Albion by 11%, but not other parameters of runner quality (Tables 2 and 3). Relationships between the density of feeder roots and runner yield were not significant for either the Fortuna or Albion cultivars.

**Table 2:** Plant parameters of harvested strawberry runners (cultivar Fortuna) treated with a seaweed extract from *Durvillaea potatorum* and *Ascophyllum nodosum* (SE) in a trial in the nursery sector at Toolangi, Victoria, Australia

Parameter	Treatment		LSD (*p* = 0.05)
	Untreated	SE	
Leaf number	4.1 a	3.8 a	0.4
Crown diameter (mm)	11.2 a	11.1 a	1.0
Petiole length (cm)	20.9 a	21.3 a	1.6
Area of the most expanded leaf (cm^2^)	67.8 a	68.9 a	5.9
Root length (cm)	21.5 a	21.5 a	1.2
Feeder roots score (1–5)^a^	2.9 b	3.5 a	0.2
Feeder root density (feeder roots cm^−1^ of structural root)	2.7 b	3.3 a	0.2

Values followed by different letters in each row are significantly different, where *p* ≤ 0.05

^a^1–5 scale described by Wing et al. (1995) where higher values mean a greater concentration of feeder roots

**Table 3:** Plant parameters of harvested strawberry runners (cultivar Albion) treated with a seaweed extract from *Durvillaea potatorum* and *Ascophyllum nodosum* (SE) in a trial in the nursery sector at Toolangi, Victoria, Australia

Parameter	Treatment		LSD (*p* = 0.05)
	Untreated	SE	
Log_10_ crown diameter (mm)	0.94 b	0.99 a	0.03
Root length (cm)	23.4 a	24.0 a	1.0
Feeder roots score (1–5)^a^	3.7 a	4.1 a	0.5
Feeder root density (feeder roots cm^−1^ of structural root)	4.3 a	4.7 a	0.5

Values followed by different letters in each row are significantly different, where *p* ≤ 0.05

^a^1–5 scale described by Wing et al. (1995) where higher values mean a greater concentration of feeder roots

There was no significant difference in soil nutrient content between treatments in the Fortuna and Albion trials (Tables 4 and 5, respectively).

**Table 4:** Chemistry at planting and harvest of soil treated with a seaweed extract from *Durvillaea potatorum* and *Ascophyllum nodosum* (SE) in a strawberry trial (cv. Fortuna) in the nursery sector at Toolangi, Victoria, Australia

Parameter	Planting			Harvest		
	Untreated	SE	LSD (*p* = 0.05)	Untreated	SE	LSD (*p* = 0.05)
pH (1:5 water)	6.5 a	6.4 a	0.3	6.6 a	6.8 a	0.5
EC (dS m^−1^)	0.13 a	0.12 a	0.04	0.25 a	0.25 a	0.04
Cl (mg kg^−1^)	13 a	12 a	2	17 a	19 a	5
Organic carbon (%)	5.2 a	5.0 a	0.5	4.8 a	4.3 a	0.7
CEC (cmol(+) kg^−1^)	12.0 a	13.0 a	2.1	12.8 a	13.7 a	3.2
Ca (cmol(+) kg^−1^)	9.5 a	10.0 a	0.9	13.0 a	13.0 a	1.2
Mg (cmol(+) kg^−1^)	1.6 a	1.7 a	0.4	3.1 a	3.5 a	0.6
Na (cmol(+) kg^−1^)	0.09 a	0.13 a	0.07	0.09 a	0.12 a	0.07
K (cmol(+) kg^−1^)	1.3 a	1.4 a	0.4	2.3 a	2.5 a	0.5
Ammonium-N (mg kg^−1^)	2.1 a	1.9 a	0.5	2.7 a	3.1 a	0.8
Nitrate-N (mg kg^−1^)	27 a	25 a	4	37 a	31 a	9
Phosphorus (Colwell) (mg kg^−1^)	250 a	270 a	52	290 a	300 a	45
Available K (mg kg^−1^)	510 a	540 a	67	690 a	600 a	105
Sulphate-S (mg kg^−1^)	20 a	16 a	7	46 a	51 a	9
Aluminium (mg kg^−1^)	0.14 a	0.19 a	0.08	0.10 a	0.12 a	0.06
Zn (mg kg^−1^)	1.3 a	1.3 a	0.2	1.8 a	1.8 a	0.2
Cu (mg kg^−1^)	0.47 a	0.48 a	0.03	0.41 a	0.57 a	0.21
Fe (mg kg^−1^)	30 a	36 a	11	50 a	52 a	9
Mn (mg kg^−1^)	1.5 a	1.5 a	0.3	1.6 a	1.9 a	0.6

Values followed by different letters in each row and each sampling time are significantly different, where *p* ≤ 0.05

**Table 5:** Chemistry at planting and harvest of soil treated with a seaweed extract from *Durvillaea potatorum* and *Ascophyllum nodosum* (SE) in a strawberry trial (cv. Albion) in the nursery sector at Toolangi, Victoria, Australia

Parameter	Planting			Harvest		
	Untreated	SE	LSD (*p* = 0.05)	Untreated	SE	LSD (*p* = 0.05)
pH (1:5 water)	6.0 a	6.0 a	0.2	5.7 a	5.9 a	0.4
EC (dS m^−1^)	0.20 a	0.19 a	0.03	0.25 a	0.22 a	0.05
Cl (mg kg^−1^)	10 a	10 a	1	16 a	14 a	4
Organic carbon (%)	4.1 a	4.5 a	0.7	4.4 a	4.8 a	0.6
CEC (cmol(+) kg^−1^)	10.2 a	9.7 a	1.9	12.6 a	10.2 a	2.7
Ca (cmol(+) kg^−1^)	7.3 a	7.9 a	0.8	9.9 a	9.7 a	0.9
Mg (cmol(+) kg^−1^)	0.9 a	0.8 a	0.2	1.0 a	1.1 a	0.3
Na (cmol(+) kg^−1^)	0.03 a	0.03 a	0.01	0.07 a	0.11 a	0.08
K (cmol(+) kg^−1^)	1.1 a	1.1 a	0.2	1.7 a	1.4 a	0.5
Ammonium-N (mg kg^−1^)	1.4 a	1.6 a	0.4	2.3 a	2.7 a	0.6
Nitrate-N (mg kg^−1^)	20 a	21 a	3	59 a	63 a	6
Phosphorus (Colwell) (mg kg^−1^)	130 a	140 a	22	210 a	250 a	51
Available K (mg kg^−1^)	440 a	440 a	19	530 a	550 a	76
Sulphate-S (mg kg^−1^)	65 a	61 a	6	86 a	90 a	7
Aluminium (mg kg^−1^)	0.24 a	0.17 a	0.11	0.28 a	0.28 a	0.10
Zn (mg kg^−1^)	0.5 a	0.6 a	0.3	0.8 a	1.1 a	0.5
Cu (mg kg^−1^)	0.20 a	0.25 a	0.09	0.35 a	0.43 a	0.26
Fe (mg kg^−1^)	24 a	30 a	9	51 a	52 a	9
Mn (mg kg^−1^)	0.8 a	0.9 a	0.3	1.6 a	1.3 a	0.6

Values followed by different letters in each row and each sampling time are significantly different, where *p* ≤ 0.05

### Strawberry fruit trial

Application of the seaweed extract in the strawberry fruit sector significantly increased commercial berry yields and revenue by 8%, compared with the untreated control (Table 6). This was equivalent to AUS$0.30 more in revenue from fruit per plant. However, there was no significant difference in fruit yield and revenue between plants only treated with the seaweed extract in the nursery sector and plants in the untreated control. Furthermore, there was no significant difference in fruit yield and revenue between plants treated with the extract in the nursery and fruit sectors, compared with plants only treated with the extract in the fruit sector. The seaweed extract had no significant effect on the individual size or proportion of commercial berries (Table 6).

**Table 6:** Commercial fruit yields and revenue of strawberry (cultivar Albion) treated with a seaweed extract from *Durvillaea potatorum* and *Ascophyllum nodosum* (SE) in the nursery and/or fruit sectors in a trial at Coldstream, Victoria, Australia

Treatment		Total fruit yield (g plant^−1^)	Total revenue (AUS$ plant^−1^)	Commercial-grade berries (%)	Fruit size (g berry^−1^)
Nursery sector	Fruit sector				
Untreated	Untreated	502.8 b	3.79 b	41.88 a	10.30 a
SE	Untreated	494.2 b	3.67 b	42.37 a	10.32 a
Untreated	SE	547.6 a	4.10 a	40.45 a	10.07 a
SE	SE	544.1 a	4.09 a	40.36 a	10.59 a
LSD (*p* = 0.05)		36.3	0.29	6.25	1.72

Values followed by different letters in each column are significantly different, where *p* ≤ 0.05

**Fig. 1:**
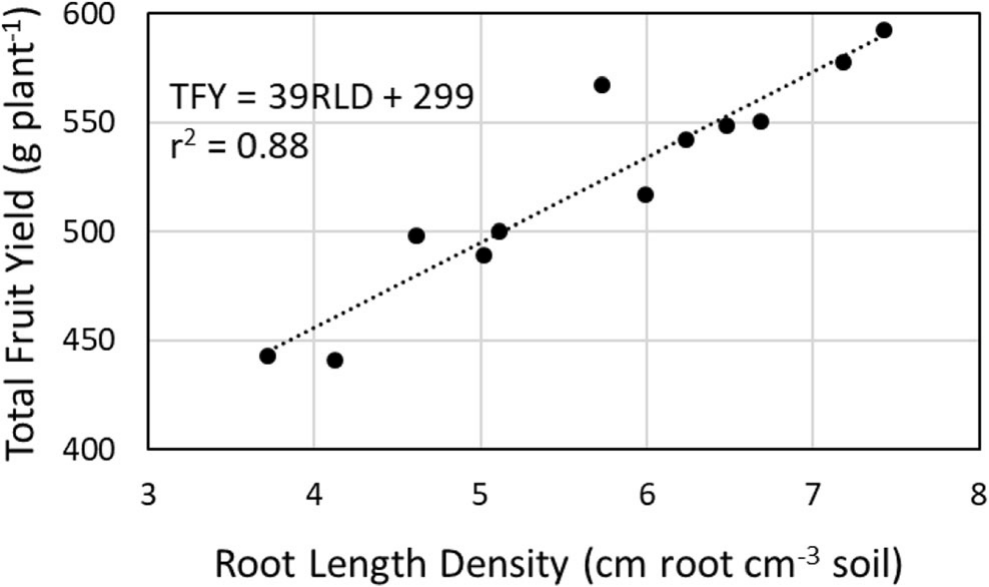
Relationship between root length density and total fruit yield of strawberry (cultivar Albion) in a trial at Coldstream, Victoria, Australia

Application of the seaweed extract in the fruit sector significantly increased root length density (root length per volume of soil) of strawberry plants by 38%, but not specific root length (root length per root mass), compared with plants in the untreated control (Table 7). Application of the extract in the nursery sector only, had no significant effect on root length density or specific root length of strawberry plants in the fruit sector. There was a significant relationship where total fruit yield increased as root length density increased (Fig. 1).

**Table 7:** Root growth at harvest of strawberry plants (cultivar Albion) treated with a seaweed extract from *Durvillaea potatorum* and *Ascophyllum nodosum* (SE) in the nursery and/or fruit sectors in a trial at Coldstream, Victoria, Australia

Treatment		Root length density (cm of root length cm^−3^ of soil)	Specific root length (cm of root length g^−1^ of root dry weight)
Nursery sector	Fruit sector		
Untreated	Untreated	4.70 b	3471.5 a
SE	Untreated	5.17 b	3317.9 a
Untreated	SE	6.55 a	3334.0 a
SE	SE	6.39 a	3346.0 a
LSD (*p* = 0.05)		36.3	319.6

Values followed by different letters in each column are significantly different, where *p* ≤ 0.05

There was no significant difference in the nutrient content of soils treated with the seaweed extract and the control at the beginning and end of the experiment (Table 8).

**Table 8:** Chemistry at planting and final harvest of soil treated with a seaweed extract from *Durvillaea potatorum* and *Ascophyllum nodosum* (SE) in a strawberry trial in the fruit sector at Coldstream, Victoria, Australia

Parameter	Planting			Final Harvest		
	Untreated	SE	LSD (*p* = 0.05)	Untreated	SE	LSD (*p* = 0.05)
pH (1:5 water)	7.2 a	7.2 a	0.2	7.1 a	7.3 a	0.3
EC (dS m^−1^)	0.19 a	0.18 a	0.03	0.30 a	0.33 a	0.05
Cl (mg kg^−1^)	27 a	30 a	4	40 a	44 a	6
Organic carbon (%)	1.4 a	1.4 a	0.3	1.2 a	1.4 a	0.5
CEC (cmol(+) kg^−1^)	11.1 a	11.4 a	0.5	12.7 a	12.6 a	0.6
Ca (cmol(+) kg^−1^)	9.4 a	10.0 a	0.8	12.0 a	12.3 a	1.0
Mg (cmol(+) kg^−1^)	1.1 a	1.5 a	0.5	1.8 a	1.8 a	0.6
Na (cmol(+) kg^−1^)	0.09 a	0.10 a	0.03	0.12 a	0.14 a	0.06
K (cmol(+) kg^−1^)	0.9 a	1.1 a	0.4	1.3 a	1.5 a	0.6
Ammonium-N (mg kg^−1^)	0.6 a	0.5 a	0.3	0.9 a	1.1 a	0.5
Nitrate-N (mg kg^−1^)	20 a	23 a	5	43 a	46 a	7
Phosphorus (Colwell) (mg kg^−1^)	140 a	160 a	34	230 a	250 a	41
Available K (mg kg^−1^)	230 a	250 a	48	380 a	410 a	63
Sulphate-S (mg kg^−1^)	41 a	55 a	18	86 a	90 a	13
Aluminium (mg kg^−1^)	0.10 a	0.10 a	0.03	0.13 a	0.13 a	0.04
Zn (mg kg^−1^)	1.3 a	1.3 a	0.3	2.8 a	2.9 a	0.5
Cu (mg kg^−1^)	0.61 a	0.48 a	0.36	0.90 a	0.96 a	0.29
Fe (mg kg^−1^)	35 a	31 a	7	86 a	93 a	11
Mn (mg kg^−1^)	3.1 a	3.2 a	0.2	3.4 a	3.7 a	0.5

Values followed by different letters in each row and each sampling time are significantly different, where *p* ≤ 0.05

## Discussion

Trials in this study consistently showed that the use of a commercial seaweed extract from *D. potatorum* and *A. nodosum* (Seasol®) has considerable potential in the strawberry industry to increase root growth, yields and revenue.

Application of the seaweed extract as a drench increased strawberry runner yields by 19% in the cultivar Fortuna and 8% in the cultivar Albion. Apart from contrasting genotypes, the difference in response to the seaweed extract between these cultivars most likely relates to their harvest times. In Australia, runners of short-day cultivars, like Fortuna, are harvested earlier (March/April) in the nursery sector than runners of day-neutral cultivars, like Albion (May/June). This is because March is the optimum time for planting runners of short-day cultivars in the fruit sector to achieve high yields (Menzel and Smith 2011, 2012). Runners harvested in the nursery sector in March can be less mature (Menzel and Smith 2011) and have less developed root systems than runners harvested later in the season. In the current trial, the ability of the seaweed extract to stimulate root growth of early-harvested runners (i.e. the cultivar Fortuna) reduced the proportion of rejected runners and markedly increased yields. In contrast, the effect of the seaweed extract on later harvested runners (i.e. the cultivar Albion), which had more developed root systems, was less pronounced. Despite the stimulatory effect on runner yields, the subsequent fruit yields of runners (cultivar Albion) treated with the seaweed extract in the nursery were no greater than those from the untreated control. This suggests that the main commercial benefit of application of the seaweed extract in strawberry nurseries is for improved runner yields and root growth, rather than improved runner performance.

The use of the seaweed extract as a drench in the strawberry fruit sector increased berry yields by 8%. This concurs with previous research that showed seaweed extracts from *A. nodosum* could increase strawberry fruit yields by up to 30% (Spenelli et al. 2010; Alam et al. 2013; El-Miniawy et al. 2014). In contrast, Boček et al. (2012) showed that a mixture of seaweed extract and amino acids did not increase strawberry fruit yields in an organic system in Europe. The seaweed extract in the trial by Boček et al. (2012), however, was only applied as a foliar treatment and was applied less frequently (five times through the cropping cycle) than in the current trial. The increase in strawberry fruit yield by the seaweed extract in the current trial also increased revenue from fruit by 8% or AUS$0.30 per plant, which is a significant return for strawberry growers compared with the cost of the treatment. In the current trial, the seaweed extract was applied at the optimum dilution, rate and method recommended by the manufacturer and established in other field trials (Mattner et al. 2013). However, it is possible that application conditions could be improved to optimise yield and revenue responses of strawberry to the seaweed extract.

Root length density and specific root length are two important parameters that measure the functionality of a plant’s root system (Zuo et al. 2004; Ostonen et al. 2007). For example, Kumar and Dey (2011) found that root length density and specific root length were highly correlated with N, P and K uptake, water-use efficiency and fruit yield in strawberry plants grown under different mulch and irrigation treatments. In the current trial, application of the seaweed extract in the fruit sector increased root length density (length of root per volume of soil) by 38%. This result is supported by direct observations that demonstrate the seaweed extract can increase root growth in strawberry (Seasol International 2017), using a time-lapse bioassay described by Arioli et al. (2015). The result also concurs with previous studies in the literature that showed that seaweed extracts from *A. nodosum* increased root length of strawberry fruit plants by up to 15% (Alam et al. 2013) and root dry weight by 35–130% (Spenelli et al. 2010; El-Miniawy et al. 2014). In contrast to root length density, application of the seaweed extract in the fruit sector had no effect on specific root length (length of root per mass of root) of strawberry plants in the current trial. The fact that application of the seaweed extract increased root length density, but not specific root length, indicates that its effect was to increase growth of the whole root system and not just finer roots with less mass (i.e. the length per gram of root was the same, but the length of root per volume of soil was greater in the seaweed extract treatment). Values for root length density and specific root length in the current experiment are broadly consistent with those reported in previous studies in strawberry (Yuen et al. 1991; Fan et al. 2011; Kumar and Dey 2011).

In the current trial, the increased root length density of strawberry caused by the seaweed extract was highly correlated (*r* = 0.94) with increased fruit yields. This discovery points to a novel mode of action provided by biostimulants containing seaweed extracts (i.e. to increase crop yield through improved functionality of the root system). Root length density and specific root length are important parameters that can define the efficiency of water and nutrient uptake by crops (Zuo et al. 2004; Ostonen et al. 2007), including strawberry (Kumar and Dey 2011). It is well established that seaweed extracts can also increase nutrient (Crouch et al. 1990; Mancuso et al. 2006; Rathore et al. 2009) and water uptake (Russo and Berlyn 1990) by plants, and their tolerance to abiotic stress such as drought (Mancuso et al. 2006; Khan et al. 2009). It is possible that seaweed extracts increase nutrient and water-use efficiency in crops through improved root length density or specific root length. This potential offers a practical agronomic way to enhance nutrient and water utilisation and improve crop tolerance to nutrient and drought stress. In this way, root length density and specific root length are important phenotypes to consider when evaluating the impact of seaweed extracts and other biostimulants on water and nutrient uptake, and crop yield.

There was no evidence from the current trials that the yield and root growth responses of strawberry to the seaweed extract were due to a nutritional effect or suppression of soil-borne disease. This is because the nutrient content of the extract is low (Wite et al. 2015), trials were conducted under conditions of high nutrition and treatment with the seaweed extract had no significant effect on soil chemistry. Furthermore, harvested roots were uniformly white across the treatments, and no soil-borne pathogens were isolated from root samples (data not shown). Rather, the seaweed extract contains a range of growth regulators and other compounds (including but not limited to cytokinins, auxins, betaines and polysaccharides) that can stimulate plant growth (Arioli et al. 2015). For example, it is well established that exogenous application of cytokinins and auxins can regulate root development and growth (Alone et al. 2006; Chapman and Estelle 2009), and various oligosaccharides contained in seaweed extracts can directly stimulate root growth (Xu et al. 2003; González et al. 2013). Therefore, we hypothesise that the plant growth regulators and other compounds contained in the seaweed extract effected the improved root growth in strawberry plants in the current trials. Further trials using plant growth regulators and other compounds in the extract as controls would add further support for this hypothesis.

Currently, the strawberry industry in most regions of the world use fumigants before planting to control soil-borne pathogens, weeds and pests (Ajwa et al. 2003; Fennimore et al. 2008; López-Aranda 2014; Mattner et al. 2014). However, many fumigants are being withdrawn due to their environmental impacts (López-Aranda et al. 2016), and this is forcing strawberry growers to consider supplementary or alternative treatments. Soil fumigation also causes an increased growth response in strawberry roots that is not related to control of pathogens and pests (Wilhelm and Paulus 1980; Porter et al. 2005). For example, Yuen et al. (1991) found that improved root density and health of strawberry plants caused by soil fumigation was correlated with increased fruit yields. In the current trials, seaweed extracts were used as supplementary treatments to soil fumigants and improved root growth of strawberry. There was a strong correlation between increased root length density caused by the seaweed extract and increased strawberry fruit yields. This suggests that use of the seaweed extract has the potential to replace or offset the increased growth response caused by soil fumigants. Further research is required, however, to evaluate whether the seaweed extract can increase strawberry root growth and yields in non-fumigated soil, and if the growth response is similar to that caused by soil fumigants. Integrated with other non-fumigant treatments that disinfest soil (e.g. biofumigation, Mattner et al. 2008; anaeorobic soil disinfestation, Shennan et al. 2014; steam, Fennimore et al. 2014; solarisation, Samtani et al. 2017), seaweed extracts or other biostimulants could form an important component of more sustainable systems to reduce strawberry growers’ reliance on soil fumigation.
